# Clinical Research: Auditory Stimulation in the Disorders of Consciousness

**DOI:** 10.3389/fnhum.2019.00324

**Published:** 2019-09-26

**Authors:** Jiajie Zhu, Yifan Yan, Wei Zhou, Yajun Lin, Zheying Shen, Xuanting Mou, Yan Ren, Xiaohua Hu, Haibo Di

**Affiliations:** ^1^International Vegetative State and Consciousness Science Institute, Hangzhou Normal University, Hangzhou, China; ^2^Department of Rehabilitation, Hangzhou Wujing Hospital, Hangzhou, China

**Keywords:** disorder of consciousness, auditory stimulation, minimally consciousness state, music, name

## Abstract

Due to the complex situation of disorder of consciousness (DOC) patients, the assessment of conscious states of these patients has become a huge challenge for a long time (Laureys et al., 2010). At present, the main clinical diagnostic method to assess the conscious state of a DOC patient is the use of a relevant behavior scale like the Coma Recovery Scale-Revised (CRS-R). In this article, we will focus on auditory stimulation and select some representative auditory stimulus, like calling names and music stimulation, to discuss the function and application of the auditory stimulus in patients with DOC and provide guidance for future research.

## Introduction

Disorders of consciousness (DOC) are commonly caused by severe brain damage, and include minimally conscious state (MCS) and unresponsive wakefulness syndrome (UWS)(Laureys et al., 2010). Patients in DOC show less awareness of the surroundings and can remain in the same stage for months or years but the difference among these patients can be found in the levels of awareness and wakefulness. While there is arousal but no awareness in UWS, MCS shows fluctuating but reproducible signs of consciousness but an absence of reliable communication (Giacino et al., 2002). Plenty of studies have been carried out and several methods have been developed to differentiate the different states, like The Sensory Modality Assessment and Rehabilitation Technique (SMART) (Gill-Thwaites, 1997), The Wessex Head Injury Matrix (WHIM) (Shiel et al., 2000), the Sensory Tool to Assess Responsiveness (STAR) (Stokes et al., 2018), the Glasgow Coma Scale (GCS) (Teasdale and Jennett, 1972), the COMA/Near Coma Scale (CNC) (Rappaport et al., 2006), the Glasgow Outcome Scale (GOS) (Teasdale and Jennett, 1972), and the Coma Recovery Scale-Revised (CRS-R).

The CRS-R is a widely used behavioral assessment scale for DOC recommended by a review of the American Congress of Rehabilitation Medicine with 6 subscales designed to assess visual, auditory, motor, and verbal, communication and arousal (American Congress of Rehabilitation Medicine et al., 2010; Gerrard et al., 2014). The CRS-R has become a standardized measure of consciousness that has been widely used for diagnostic assessment in studies involving persons with DOC (Gerrard et al., 2014).

The primary auditory cortex is made up of horizontal gyrus and superior temporal gyrus, and the afferent fibers of the auditory nerve form the lateral thalamus after crossing the same side of the brain stem, then the lateral thalamus fibers reach the medial geniculate body, and the latter emits fibers to form auditory radiation ending in the primary cortex. Middle ear, inner ear, or auditory nerve damage may lead to hearing disorders (Allen et al., 2013). Auditory stimulation is a kind of stimulation that can enrich the environment to improve arousal and awareness state in patients with DOCs and is widely used. Clinical studies believe that the generation of consciousness is the result of the joint action of multiple brain networks. The auditory cortex is an important part of some of the brain networks (Default mode network) (Cook et al., 2019). Clinically, the activation of the auditory cortex is compared with the patient’s state of consciousness and it is concluded that the retention of the auditory cortex may be an indicator of the retention of consciousness (Heine et al., 2015). Tests based on auditory stimulation like auditory startle and localization to sound in CRS-R (American Congress of Rehabilitation Medicine et al., 2010) play an important role and more auditory stimulations have been used on other subjects (Teasdale and Jennett, 1972; Gill-Thwaites, 1997; Shiel et al., 2000; Stokes et al., 2018). Auditory stimulation is also effective in the recovery of consciousness, and a recent study focusing on the impact of sensory stimulation programs (SSP) in the recovery of DOC highlighted that SSP may not be sufficient to restore consciousness but it may lead to improved behavioral responsiveness in MCS patients (Cheng et al., 2018).

## Auditory Stimulus in Doc for Diagnosis

Though auditory stimulation plays an important role, patient’s response to stimuli like music, bells, and calling names can differ, therefore researchers tried to select the most sensitive stimulation for diagnosis between auditory stimulus and auditory and other sensory stimulation, while behavioral indicators (sound fixation, CRS-R scores), brain neurophysiological indicators (fMRI, EEG, ERP), and other indicators like skin conductance level (SCL) were applied to evaluate the effect. Due to the diversity of assessment methods, the selection of methods is particularly important. Active paradigms can be used to compare patients and healthy controls, if they are activated in the related region in the active paradigm fMRI. Some patients with higher levels of consciousness were even able to answer simple yes or no questions by completing different tasks. Active paradigms are also helpful to recognize conscious patients who were misdiagnosed on the behavior scale, and couldn’t answer the CRS-R but achieved positive results in the active paradigms fMRI (Naci and Owen, 2013). A study aiming to find the difference in SCLs during music stimulus and odor stimulus showed that no obvious difference was observed between two conditions (Li et al., 2018), while other studies with the same type of stimulation but employing different dependent measures like behavior change came out with totally different results (Heine et al., 2017) (Please see Table 1, the reference has been summarized in it). In consideration of the outcome of these studies, the problem may not be the choice of stimulus but the SCL is not an appropriate indicator for the study, which highlighted that selecting a suitable measure is the key point in DOC researches and its role in diagnosis of DOC.

**TABLE 1:** A review of the literature related to this article.

**Objectives**	**References**	**Methods**	**Stimulus**	**Subjects**	**Main outcome**
Diagnosis	Li et al., 2018	EEG	Music, call-names, habit stimulation	9 MCS; 10 UWS	Call-name stimulation > habit > music stimulations.
	Heine et al., 2017	CRS-R	Preferred or neutral auditory stimuli and odor stimuli	7 MCS; 6 UWS	Auditory stimuli > a olfactory stimuli
					Preferred stimuli > neutral stimuli.
	Verger et al., 2014	CRS-R	Preferred music, a continuous sound	5 MCS+; 1 MCS−	Preferred music > meaningless sound
	Cheng et al., 2013.	Behavior	Patient’s own name, ringing bells	39 MCS; 47 UWS	Own name > ringing bells
	Wu et al., 2018.	EEG	Music, own names, white noise	7 MCS; 7 UWS	Subject’s own name > music
					Subject’s own name > white noise
					White noise/music > silence
					No obvious difference observed between white noise and music.
	Bekinschtein et al., 2004.	fMRI	Non-familiar voice, silence, mother’s voice	Case report	Familiar voice > silence
					Familiar voice > unfamiliar voice
	Okumura et al., 2014.	fMRI	Baseline sound stimulation; instrument sound stimulation	5 VS; 2 MCS; 21 healthy controls	MCS > VS [bilateral superior temporal gyri (STG)]
	Luaute et al., 2018.	SCL	Preferred music, neutral sound, preferred odors, and neutral odors.	7 MCS; 6 UWS; 7 healthy controls	No significant difference between conditions was detected in patients.
	Kempny et al., 2018	ERP	Subject’s own name and other’s name	12 HC; 5 VS; 11 MCS	Using this paradigm in 4 DOC patients we detected a statistically significant difference in EEG response to their own name versus other peoples’ names.
Prognosis	Di et al., 2007.	fMRI	Subject’s own name	7 VS; 4 MCS; 12 health	Adults and patients in an MCS.
	Wang et al., 2015.	fMRI	Subject’s own name	39 MCS (23 non-traumatic; 16 traumatic)	12 out of 16 VS/UWS patients with higher level activation recovered to MCS or EMCS, whereas 17 out of 23 VS/UWS patients with no activation or activation.
	Fischer et al., 2008	A passive oddball paradigm (SON)	Subject’s own name	50 coma	Compared to MMN, P3 showed as large a specificity for awakening.
	Perrin et al., 2006.	ERP-P3	Subject’s own name	5 VS; 6 MCS; 4 LIS	A P3 component was observed in response to the patient’s name in LIS, in all MCS patients, and in 3 of 5 patients in a VS.
	Qin et al., 2008	ERP-oddball; CRS-R	Subject’s own name	4 coma	N100 9/13
	Qin et al., 2008 Literature	ERP-oddball; CRS-R Methods	Subject’s own name Stimulus	7 VS	Nd 7/13
				2 MCS	MMN 1/13
					P300 4/13
					2 MCS has Nd and N100, but no MMN and P300
				Subjects	Main outcome
	Berlad and Pratt, 1995	Active and passive oddball 3-word paradigm	Subject’s own name	12 subjects	P300 amplitude to the subject’s name was larger than to the irrelevant rare word in 9 of the 10 subjects.
	Heine et al., 2015	fMRI	Five musical excerpts selected from a questionnaire	4 MCS; 3 UWS; 8 healthy participants	Music > control (left precentral gyrus and the left dorsolateral prefrontal cortex)
	Magee et al., 2014.	MATADOC; SMART	/	21 patients	MATADOC = SMART (the principal subscale)
	O’Kelly et al., 2013	EEG; behavioral responses	Preferred music; improvised music; disliked music; white noise; silence	12 VS; 9 MCS 20 healthy controls	Frontal midline theta in 6 VS and 4 MCS subjects, and frontal alpha in 3 VS and 4 MCS subjects were found during preferred music stimulation
Treatment	Ribeiro et al., 2014.	Vital signs; Facial expressions	Musical stimuli; radio; CRM; RMNS	26 VS	Music stimuli > radio/CRM/RMNS
	Raglio et al., 2014.	Physiological parameters; behavioral responses	Individual AMT	4 MCS; 6 VS	After therapy > before therapy
	Steinhoff et al., 2015	PET	Music Therapy (MT)	4 VS	MT > control (frontal, hippocampal, and cerebellar region of the brain)
	Park et al., 2016	The reduction of agitation	Preferred music	14 patients	Preferred music > control
	Sun and Chen, 2015	EEG; GCS	Music Therapy (MT)	40 patients	Music group > control group
	Magee et al., 2016	MATADOC	/	21 patients	MATADOC subscales two = MATADOC subscales three
	Magee et al., 2015	MATADOC; CRS-R; CNC; PCC	/	4 patients (children)	MATADOC > others (auditory and visual)
	O’Kelly and Magee, 2013	MATADOC; SMART	/	42 patients	MATADOC > SMART (auditory and visual); SMART > MATADOC (motor)

*“>” means that there is a better or more significant response to the corresponding stimulus or methods. “=” means that there is a equal response to the corresponding stimulus or methods. UWS, unresponsive wakefulness syndrome; MCS, minimally conscious state; CRS-R, Coma Recovery Scale-Revised; GCS, the Glasgow Coma Scale; CNC, the COMA/Near Coma Scale; SMART, The Sensory Modality Assessment and Rehabilitation Technique; MATADOC, Music therapy assessment tool for awareness in disorders of consciousness; PCC, The Pediatric Center Criteria for Diagnosing a Persistent Vegetative State; ERP, event related potentials; SCL, skin conductance level; EEG, Electroencephalogram; fMRI, functional magnetic resonance imaging; RMNS, relaxing music with nature sounds; CRM, classical relaxing music; AMT, active music therapy.*

Besides measures, choosing a reliable stimulus is important. From the studies we gathered, it appears clear that compared to other stimulation, auditory stimulation has a potential role in improving state of consciousness and it’s worth noting that calling a patient’s name might be the most effective choice followed by preferred music. A comparison between calling a patient’s name and other auditory stimulations varying from white noise to tones has been carried out through different dependent measures in recent years (Cheng et al., 2013; Verger et al., 2014; Heine et al., 2015; Li et al., 2018; Wu et al., 2018), and the results seemed to confirm the conclusion that calling the Subject’s Own Name (SON) may be the most effective auditory stimulation. An EEG study ran by Kempny et al. (2018) using SON and others’ name on 16 patients (5 UWS; 11 MCS) indicated that there seemed to be no significant difference between DOC patients’ response to SON and others’ name at the group level, but 4 of 15 patients showed significantly different responses between hearing their own name and others’ names at a single subject and two of these patients had similar responses as controls (Kempny et al., 2018), while another fMRI study applying the patient’s mother’s voice and a stranger’s voice to read the same story reported by Bekinschtein et al. (2004) showed that, compared to the unfamiliar voice, the brain activation is stronger under a familiar sound condition. The patient’s mother’s voice activates the extended amygdala, an emotionally related structure, and a directly connected area such as the insula, perhaps acting jointly as limbic integration cortex.

Combining this result with other studies’ (Bekinschtein et al., 2004), it’s safe to say a personal-relevant stimuli works better than a meaningless sound, but personal-relevant includes not only SON but also stimuli like preferred music or similar. Studies also showed that compared to meaningless sound or unlike music, the auditory network of DOCs seemed to have stronger functional connectivity while hearing one’s preferred music than the control condition, and especially in the left precentral gyrus and the left dorsolateral prefrontal cortex (Heine et al., 2015), functional connectivity of the external network was also enhanced during the music palying in the temporo-parietal junction (Heine et al., 2015). The same conclusion was reached by another CRS-R study (Verger et al., 2014).

For its unique role as a personal-relevant stimulus and the lack of sensitive assessment in auditory block in CRS-R, “Music therapy assessment tool for awareness in disorders of consciousness” (MATADOC) has been developed as a more detailed auditory assessment tool (Magee et al., 2014) based on three subscales, the “Principal Subscale”, the “Musical Parameter and Behavioral Response Type” and the third subscale, “Clinical Information to Inform Goal Setting and Clinical Care” (Magee et al., 2014). Only the “Principal Subscale” achieved diagnosis and the other two subscales are designed to guide treatment (Magee et al., 2016). MATADOC could serve as a better way for the pediatric population suffering from DOC given the lack of language and motor functions (Magee et al., 2015).

The selection of the stimulus is crucial for an accurate evaluation of the state of patients with disorders of consciousness as it determines the level of processing the patient can achieve with the stimulation from his/her environment, and a personal-relevant stimulus works better than an irrelevant stimulus, therefore calling a subject’s name could be the best choice of the diagnosis of DOC patients. It must be noted that there is no consensus on how to clinically assess the localization to sound in patients recovering from coma.

## Auditory Stimulation for Prognosis

“Calling one’s name” has been incorporated into the latest guidelines for disorders of consciousness in the United States. Family members often call the patient’s name during the nursing process and there is a term in psychology called the “cocktail party effect” (Wood and Cowan, 1995), which refers to the fact that if someone calls your name in a very noisy environment (such as a cocktail party), you will hear it first, indicating that the act of calling someone’s name can enter consciousness before other auditory stimuli. This phenomenon also exists in patients with consciousness disorders, and can represent the most effective stimulus. Calling the patient’s name has become a staple in the field of international consciousness disorder research and has been widely used in clinical practice. In addition, it has the significant advantage of eliciting some behavioral responses compared to the stimulation of other auditory channels such as bells (Naci and Owen, 2013).

As early as in Di et al. (2007) found out that when hearing a familiar voice calling their names, two patients experienced VS joint temporal lobe area in the brain (higher level of auditory processing area) activation, while other patients just showed activation in the primary auditory cortex. The two patients mentioned before regained consciousness after 3 months. Another research showed that in a passive condition, alpha desynchronization was observed during familiar voice and name stimuli in the right hemisphere (del Giudice et al., 2014). Working with a leading international team, patients were asked to imagine a scene during which they imagine a scene during an imaginary scene. The study found that the etiology of patients can further improve the accuracy of prognosis prediction. Traumatic VS patients with auditory cortical activation were more likely to recover consciousness (92.3%). Non-traumatic VS patients without this activation were more likely to have a poor prognosis (85%) (Wang et al., 2015).

According to EEG studies, hearing a subject’s own name induces the positive component of event-related potential and beta power suppression (Tamura et al., 2016). SON as a novel feature in an MMN design can be utilized to increase the prognostic value of ERPs in comatose patients and to assess unconscious cognitive processes in uncommunicative patients (Fischer et al., 2008). And the presence of a novelty P3 in response to the subject’s own first name presented as a novel stimulus has shown a good correlation with coma awakening (Morlet and Fischer, 2014).

Calling patients’ names is not merely a kind of stimulant commonly used by family members, but also more likely to result in self-consciousness and social consciousness. It has been shown to have a prognostic effect, indicating an increased chance of recovery within 12 months (Giacino et al., 2018b).

## Auditory Stimulation for Treatment Measure

As proven by several studies, music is a useful auditory stimulation that plays a fundamental role in DOCs. Here, we will discuss its use in DOC therapy.

A fMRI study carried out by Okumura in 2014 (Magee et al., 2014) showed that the activation of bilateral STG (superior temporal gyri) in patients with MCS was different than in VS patients during three different tasks with three types of sounds, such as a baseline sound stimulation, an instrument sound stimulation and a music stimulation. The results indicated that, compared to meaningless sound, brain activation is easier to be observed during MS (Okumura et al., 2014). Another study on functional connectivity showed that, compared to generic music, the auditory network showed stronger functional connectivity in the left precentral gyrus and the left dorsolateral prefrontal cortex during the listening to the patient’s preferred music, and the functional connectivity of the external network was also enhanced in the temporo-parietal junction (Heine et al., 2015). Perrin et al. proposed that the reason for the positive effects of preferred music on arousal and attention observed in DOC is that the music prompts the activation of two anticorrelated brain networks for internal and external engagement at the same time, unlike the usual pattern, and their theory explained the findings in other similar studies (Perrin et al., 2015).

Though evidence is still limited, neuroimaging studies have shown that music listening activates a list of bilateral networks related to attention, memory and sensory system (O’Kelly et al., 2013; Rollnik and Altenmuller, 2014), and given its ability to boost cognition in DOC, music is a useful tool. It potential role in the treatment of DOCs has been highlighted in recent years, and behavioral and physiological methods have been applied to verify its reliability (Raglio et al., 2014; Ribeiro et al., 2014). But only recently brain imaging methods like fMRI, PET, EEG have been introduced in the research about music therapy in DOCs. A study employing PET as a reliable measure to better understand the relationship between music therapy and neuroscience before, during and after music therapy (Steinhoff et al., 2015) scanned the metabolism of the brain three times with PET during resting state, first exposure to music therapy (MT), and last exposure to MT. The final result showed that in the three areas analyzed in this study, the frontal areas, the hippocampus and the cerebellum, patients in music therapy showed higher brain activity than the control group (Steinhoff et al., 2015). The same conclusion was carried out in three other EEG studies (O’Kelly et al., 2013; Sun and Chen, 2015; Park et al., 2016), one of which aimed at evaluating the effects of a preferred music intervention on the reduction of agitation in TBI patients, showing that patients in music therapy also had better behavioral or neurophysiological indexes than the control group, and that a significantly greater reduction in agitation was observed in the patients listening to the preferred music (Park et al., 2016). These results stated that music therapy can prompt the activation of related brain regions, promote the recovery of patients’ behavior and improve the conscious state of DOCs (Okumura et al., 2014; Rollnik and Altenmuller, 2014; Heine et al., 2015; Perrin et al., 2015), and that personally relevant stimuli like preferred music are more effective. This is consistent with the conclusion drawn from the studies listed above (Cheng et al., 2013; Verger et al., 2014; Heine et al., 2015).

For its unique role as a personally relevant stimulus and the lack of sensitive assessment in auditory block in CRS-R, the “Music therapy assessment tool for awareness in disorders of consciousness” (MATADOC) has been developed as a more detailed auditory assessment tool (Magee et al., 2014) based on three subscales, the “Principal Subscale”, the “Musical Parameter and Behavioral Response Type” and the third subscale, “Clinical Information to Inform Goal Setting and Clinical Care” (Magee et al., 2014). Only the “Principal Subscale” is a diagnosis tool, while the other two subscales are designed to guide treatment (Magee et al., 2016). MATADOC could serve as an effective tool for the pediatric population suffering from DOCs, given the lack of language and motor functions (Magee et al., 2015).

Though music is not as promising as calling the patient’s name in diagnosis, as a well-worked auditory stimulation, its application in the treatment of DOCs has been widely shown to be useful, but more neurophysiology and behavioral support is also required to figure out the exact connection between music therapy and the recovery of consciousness.

## Discussion

As a kind of sensory stimulation, auditory stimulation can efficiently provide a good cognitive environment for patients suffering from acute brain injuries, helping patients recover and serve as a diagnostic tool or reference to judge patients’ states of consciousness, and its role is increasingly known by the public in terms of treatment and diagnosis (O’Kelly et al., 2013; Rollnik and Altenmuller, 2014). As the strongest personal-related stimulus, Calling patients’ names can effectively activate the related brain regions, and the difference between MCS and VS can be seen through imaging techniques like fMRI or PET (the studies involved are listed above in this paper) (Perrin et al., 2006; Di et al., 2007; Qin et al., 2008; Cheng et al., 2013). fMRI studies assessing brain activation to the patients’ names have reported the activation of self-related brain regions depending on the level of consciousness in patients recovering from coma (Di et al., 2007; Cheng et al., 2013) and compared to music, hearing names seem to be a more promising stimulation to boost cognition, as proven by the fact that cerebral activation was higher in patients stimulated by their own name, especially in the temporal lobe (Wu et al., 2018). As for music, it’s more like a treatment. O’Kelly et al. integrate music stimulation with other sensory stimulations to make a music-based scale, the function of which is mainly guiding treatment (Magee et al., 2014, 2016), while some researches have shown that personal related stimulation provides a better cognition state (Wu et al., 2018), and, compared to preferred music, normal music has a weaker improvement in DOC.

But it is difficult to employ sound stimulation as a sufficient tool of diagnosis, prognosis and treatment. First, the choice of sound is very subjective. The definition of personal related stimulus is not so clear, making it difficult for doctors to choose the most suitable sound stimulation (Wu et al., 2018), although the name is now clinically considered to be one of the best sources of stimulation and the application of the name is extensive and effective in diagnosis and prognosis, but its role in the treatment is not as significant as music (Sun and Chen, 2015). The author speculates that this may be related to the simple pronunciation of the name, the single content, and the tone of the calling, volume, etc. Music is relatively rich in content and can be well controlled in volume. A varied source of stimulation may do well in improving the patient’s state of mind, but how to choose the music is a challenge. At present, the choice of music is largely based on the patient’s family’s judgement and the popularity of the music, which increases the uncertainty of the treatment efficacy, as the diagnosis of children with mental disorders based on music shows. The second problem is in the diagnostic method. At present, the most credible method is the medical imaging. However, the operation is cumbersome. A diagnosis often takes a long time to prepare and analyze, and it costs more. This is detrimental to the accurate treatment of patients, and it is not the most appropriate choice for some patients. Third, given the number of samples and neuroanatomical evidence, the current sample number of studies is not high enough to promote auditory stimulation as an effective diagnostic tool. At present, the studies on the relationship between hearing and consciousness are mostly carried out on rats and non-patients under general anesthesia. Auditory consciousness has been well studied in humans, and auditory cortical neuron processing has also been well studied in animal models (Eagleman and MacIver, 2018). However, the latest experimental results show that there is no relationship between the activity of primary auditory cortex and the loss of conscious activity in rats under general anesthesia (Eagleman and MacIver, 2018), and the generation of consciousness is complex. The reason why auditory stimulation was chosen as an indicator is that some patients cannot show clinical behavior to the stimulation but their retention of consciousness can be observed in imaging. This part of the patient’s cortex response to sound stimulation is similar to that of healthy people (Okumura et al., 2014). But the neuroanatomical basis of the connection of auditory cortex and consciousness generation has not yet been discovered. The activation of the auditory cortex cannot diagnose the patient’s state of consciousness alone.

## Conclusion

In conclusion, calling the patient’s name is a better choice than music as an effective auditory stimulation, but music has its unique role in treatment. Auditory stimulation still cannot be an independent diagnostic tool, and its value in diagnosis needs to be matched with other sensory stimulation. The diagnosis of DOC requests multidimensional assessments. The relationship between auditory stimulation and consciousness is still unknown. Future studies need to be more precise, focusing on the connection between auditory stimulation and consciousness in neurology with a larger sample.

## Author Contributions

JZ, YY, and YL contributed to the conception and design of the study. JZ and YL organized the database and wrote the first draft of the manuscript. ZS, XM, and YR wrote the sections of the manuscript. All authors contributed to the manuscript revision and read and approved the submitted version.

## Conflict of Interest Statement

The authors declare that the research was conducted in the absence of any commercial or financial relationships that could be construed as a potential conflict of interest.
